# Birth-dose hepatitis B vaccine and bronchopulmonary dysplasia risks in preterm infants

**DOI:** 10.2471/BLT.24.293032

**Published:** 2025-03-01

**Authors:** Matthew F Daley, Jason M Glanz

**Affiliations:** a Institute for Health Research, Kaiser Permanente Colorado, 16601 E. Centretech Parkway, Aurora, CO 80011, United States of America.

The World Health Organization (WHO) recommends hepatitis B virus (HBV) vaccine within the first 24 hours of life regardless of birthweight or gestational age;^1^ however, real-world safety data for birth-dose HBV vaccination among preterm and low birthweight infants are limited. Preliminary data suggest that T-helper lymphocyte type 2 immune response in preterm infants may be associated with bronchopulmonary dysplasia, a chronic lung disease, and that delayed birth-dose HBV vaccination may be associated with an attenuated T-helper lymphocyte type 2 immune response.^2^

In a study in extremely premature infants in Australia, no association between receiving HBV vaccine at birth and bronchopulmonary dysplasia, was found.^3^ This study provides additional reassuring evidence about the safety of HBV vaccines, particularly in newborns. By linking several data sources from Victoria, Australia, the authors identified 818 infants born before 29 weeks estimated gestational age during the three-year study observation period. Among these infants, 37.4% (306/818) received a birth dose of HBV vaccine and 62.6% (512/818) did not. The authors observed that 50.7% (155/306) of HBV vaccine-exposed infants and 61.9% (317/512) of unexposed infants developed bronchopulmonary dysplasia.^3^ In the primary adjusted analyses, receipt of a birth dose of HBV vaccine was not associated with increased incidence of bronchopulmonary dysplasia.^3^ In secondary analyses, there was no association between birth-dose HBV vaccination and all-cause mortality in the first 3 months of life.^3^

While variability in HBV vaccine exposure created an opportunity to test HBV vaccine safety hypotheses, it also reflects the challenges with using observational data for vaccine safety research. Vaccinated individuals often differ from the unvaccinated by important factors unmeasured in electronic health data sources.^4^ The authors acknowledged this risk of confounding by contraindication.^3^ Infants born before 29 weeks estimated gestational age require intensive medical support immediately after birth, generally including respiratory, haemodynamic and temperature support, as well as other interventions.^5^ Although WHO recommends HBV vaccination within the first 24 hours of life for all infants,^1^ other high priority medical interventions may take precedence for infants requiring intensive care. Treating clinicians and parents may also have concerns about the safety and effectiveness of vaccination in medically fragile extremely preterm infants.^6^ A healthy vaccine recipient effect can result, whereby those vaccinated are in better health at the time of vaccination than the unvaccinated, thus masking a potential association between vaccination and an adverse event.^4^ In the current study, several of the known risk factors for bronchopulmonary dysplasia (such as length and intensity of mechanical ventilation, degree of oxygen support and post-natal infections)^7^ could have been less prevalent among the vaccinated than the unvaccinated infants. While the authors attempted to adjust for underlying disease risk using the data available, the potential for bias remained.

When confounding by contraindication is anticipated in vaccine safety studies, considering alternative study designs to mitigate the risk of bias is important. Given the importance of timely administration of birth-dose HBV vaccine for disease prevention,^1^ randomizing infants to immediate versus delayed HBV vaccination would not be feasible or ethical. Self-controlled designs have been used in observational vaccine safety studies to address confounding by contraindication: within an individual, incidence of an adverse event in a risk window after vaccination is compared to incidence in a control window more distant in time from vaccination.^8^ Because individuals are compared to themselves, unmeasured, fixed confounding variables are controlled for by proxy. In the current study, applying a self-controlled design would be difficult because control time before vaccination (that is, before birth) is not available. Furthermore, it takes weeks to months for bronchopulmonary dysplasia to develop and time-varying confounding may be present. The solution may be prospective cohort studies with primary data collection, to enable more complete capture of covariate information including the length and intensity of mechanical ventilation.

Finally, recognizing that the study design and data challenges in the Australian Vaccine Safety Health Link are similar to other vaccine safety surveillance systems globally is important.^9^ Accordingly, the current study highlights important areas for future research and development. As new products are developed to prevent infectious diseases in infants, including infant vaccines, monoclonal antibodies^10^ and maternal vaccination, additional investment is needed in expanding data linkages to evaluate medical product safety in newborns.
